# Microstructural Changes within the Basal Ganglia Differ between Parkinson Disease Subtypes

**DOI:** 10.3389/fnana.2016.00017

**Published:** 2016-02-23

**Authors:** Lidia M. Nagae, Justin M. Honce, Jody Tanabe, Erika Shelton, Stefan H. Sillau, Brian D. Berman

**Affiliations:** ^1^Department of Radiology, University of Colorado Anschutz Medial CampusAurora, CO, USA; ^2^Department of Neurology, University of Colorado Anschutz Medial CampusAurora, CO, USA; ^3^Neurology Section, Denver VA Medical CenterDenver, CO, USA

**Keywords:** Parkinson disease, motor subtypes, diffusion tensor imaging, basal ganglia

## Abstract

Diffusion tensor imaging (DTI) of the substantia nigra has shown promise in detecting and quantifying neurodegeneration in Parkinson disease (PD). It remains unknown, however, whether differences in microstructural changes within the basal ganglia underlie PD motor subtypes. We investigated microstructural changes within the basal ganglia of mild to moderately affected PD patients using DTI and sought to determine if microstructural changes differ between the tremor dominant (TD) and postural instability/gait difficulty (PIGD) subtypes. Fractional anisotropy, mean diffusivity, radial, and axial diffusivity were obtained from bilateral caudate, putamen, globus pallidus, and substantia nigra of 21 PD patients (12 TD and 9 PIGD) and 20 age-matched healthy controls. *T*-tests and ANOVA methods were used to compare PD patients, subtypes, and controls, and Spearman correlations tested for relationships between DTI and clinical measures. We found our cohort of PD patients had reduced fractional anisotropy within the substantia nigra and increased mean and radial diffusivity within the substantia nigra and globus pallidus compared to controls, and that changes within those structures were largely driven by the PIGD subtype. Across all PD patients fractional anisotropy within the substantia nigra correlated with disease stage, while in PIGD patients increased diffusivity within the globus pallidus correlated with disease stage and motor severity. We conclude that PIGD patients have more severely affected microstructural changes within the substantia nigra compared to TD, and that microstructural changes within the globus pallidus may be particularly relevant for the manifestation of the PIGD subtype.

## Introduction

Heterogeneous clinical phenotypes such as tremor dominant (TD) and postural instability/gait difficulty (PIGD) have long been recognized in Parkinson disease (PD) (Jankovic et al., 1990). These PD motor subtypes differ in their clinical course, with the PIGD subtype generally having a more severe course and greater association with non-motor symptoms (Burn et al., 2006; Rajput et al., 2009; van Rooden et al., 2011; de Lau et al., 2014). Improvement in motor function following deep brain stimulation (DBS) may also differ between these subtypes (Katz et al., 2015). Refinement in the clinical classification of PD subtypes is an important goal in PD research to better understand risk factors, mechanism of disease, underlying genetics, and clinical course, as well as to inform better treatment strategies.

Diffusion tensor imaging (DTI), classically used to probe water motion at the cellular level to investigate white matter integrity, can be used to investigate gray matter microstructure *in vivo* (Schwarz et al., 2013). The gray matter structure of greatest interest in PD has been the substantia nigra (SN) (Chan et al., 2007; Vaillancourt et al., 2009; Zhan et al., 2012; Zhang et al., 2015). In general, decreased fractional anisotropy (FA) along with increased diffusivity measures including mean diffusivity (MD), axial diffusivity (AD), and radial diffusivity (RD) of the SN has demonstrated promise in being able to distinguish PD patients from healthy controls. These prior reports, however, have also shown variable and at times conflicting results (Chan et al., 2007; Vaillancourt et al., 2009; Schwarz et al., 2013).

In PD, few DTI studies to date have investigated microstructural changes affecting basal ganglia beyond the SN. In a study by Kim et al. increased MD in the caudate, putamen, and globus pallidus in the absence of significant FA changes was found in a cohort of PD patients (Kim et al., 2013). In a study by Prodoehl et al. FA and diffusivity measures in the caudate, putamen, globus pallidus, as well as SN were found to have the ability to discriminate PD from controls as well as PD from atypical parkinsonism and essential tremor (Prodoehl et al., 2013). These studies suggest that the microstructure in a variety of subcortical brain regions may be differentially affected in PD and atypical parkinsonian disorders.

In the present study, we utilized DTI to investigate microstructural changes within the basal ganglia, including the caudate, putamen, globus pallidus and SN, in PD patients and healthy controls. Our objectives were to: (1) investigate changes in DTI measures (FA, MD, AD, and RD) that occur in PD within the basal ganglia beyond just the substantia nigra, and (2) determine if the DTI measures obtained from these structures differ between the TD and PIGD motor subtypes of PD when compared to controls. We further tested whether DTI measures within the basal ganglia correlated with clinical disease characteristics including duration, stage and severity.

## Materials and methods

### Subjects

Twenty-one patients diagnosed with idiopathic PD according to the UK Parkinson Society Brain Bank criteria (Hughes et al., 1992; 12 male; 61.1 ± 7.7 years) and 20 age-matched healthy controls (11 male; 61.1 ± 9.0 years) were recruited. Patients were Hoehn & Yahr (H&Y) stage I-III and known to be responsive to dopaminergic medication. TD and PIGD scores were calculated using the Movement Disorder Society Unified Parkinson Disease Rating Scale (MDS-UPDRS) III motor scores for patients off medication according to previously reported methods (Stebbins et al., 2013). TD/PIGD ratios were generated at the time of screening and subtypes assigned using a cutoff score of ≥1.15 for TD and ≤0.90 for PIGD; scores >0.9 and <1.15 were classified as indeterminate. Indeterminate patients were excluded from the study. Of the PD group, 12 were TD and 9 were PIGD. All patients were evaluated using the total MDS-UPDRS for clinical staging, the Montreal Cognitive Assessment (MoCA) for cognitive evaluation, and the Beck Depression Inventory (BDI) and Neuropsychiatric Inventory (NPI) for identification of psychiatric comorbidities. The study was approved by the local Institutional Review Board and patient consent followed the principles of the Declaration of Helsinki.

### DTI imaging acquisition

MR imaging was performed on a 3T Signa HDxt MRI (GE Medical Systems, Milwaukee, WI) with an 8-channel brain phased- array head coil. 32-direction diffusion imaging was acquired with a spin echo, echo planar imaging (EPI) sequence using the following parameters: FOV: 26 × 26 cm, TR/TE: 16000/92 ms, matrix size: 128 × 128, slice thickness: 2 mm, and *b*-values of 0 and 1000 s/mm^2^.

### Image analysis

Image analysis was performed using the FMRIB Software Library tools (FSL) (www.fmrib.ox.ac.uk/fsl). Source diffusion data was initially pre-processed to remove the effects of eddy current distortions with the FMRIB's Diffusion Toolbox (FDT). Using the FMRIB's DTIfit tool, the eddy current corrected diffusion data for each subject was processed to generate voxel-wise maps of FA, MD, AD, and RD. The FMRIB's Linear and Non-Linear Image Registration Tools (FLIRT followed by FNIRT) were then used to normalize each subject's FA maps to 1 × 1 × 1 mm^3^ Montreal Neurological Institute (MNI) 152 standard space using the FMRIB58-FA template as the target. This non-linear transformation was performed to allow calculation of inverse normalizations (using the *invwarp* tool) for transposition of ROI masks to subject space as described below.

### ROI masks

Using the Harvard-Oxford cortical and subcortical structural probabilistic atlas provided in FSLView, masks were extracted from the labels for the right and left caudate nucleus, putamen and globus pallidus. The probabilistic masks for each structure were was then thresholded using the *fslmaths* tool to a probability of 80% to decrease the possibility of overlap with adjacent structures. As standard atlases such as the Harvard-Oxford atlas lack labels for the SN, the Atlas of the Basal Ganglia (ATAG) MNI 04 atlas (Keuken et al., 2014) was used to extract SN masks. First the atlas was downsampled from 0.4 × 0.4 × 0.4 to 1 × 1 × 1 mm^3^ in MNI 152 space. Then, in a similar fashion to the caudate, putamen and globus pallidus, FSLView was then used to extract the standard space mask for each SN and *fslmaths* was used to threshold the resulting masks to 50%.

### ROI transposition to subject space and quality assurance

**Figure 1:**
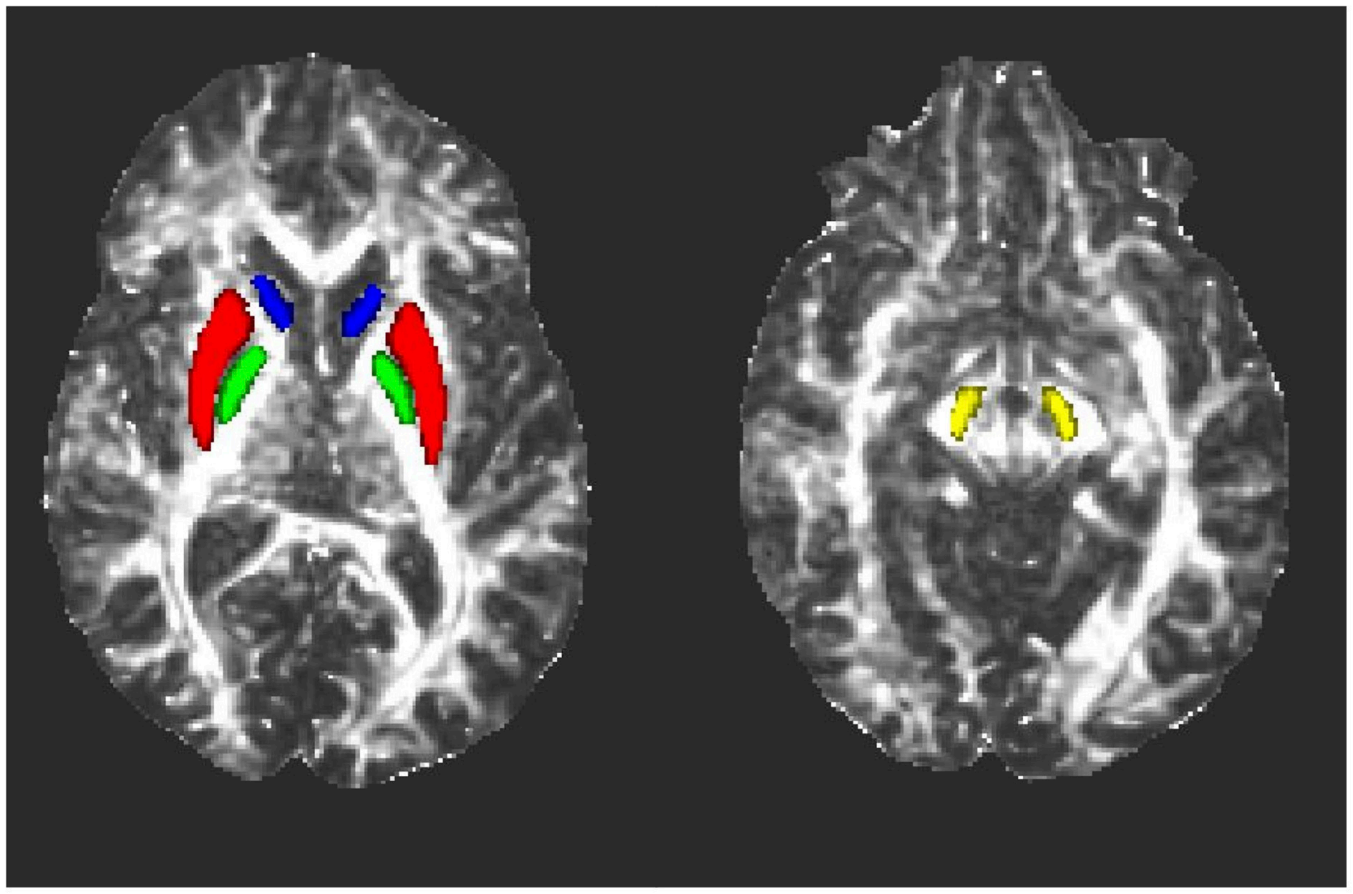
**Representative axial fractional anisotropy (FA) maps from one subject showing the locations of the ROIs after transformation to subject space**. Blue, caudate nucleus; red, putamen; green, globus pallidus; yellow, substantia nigra.

The FMRIB's *invwarp* tool was used to calculate the inverse warp of the previously described non-linear transformation of the FA maps to standard space. This inverse transformation was then used with the *applywarp* tool to de-normalize all eight thresholded, standard space masks to the subject space for each study participant. This was performed so that all data evaluation could be done in subject space to avoid possible data modification by the normalization process. A blinded quality assessment review was then performed by a board certified neuroradiologist (J.M.H.), in which the ROIs in subject space were superimposed on each subject's FA map to confirm the final accuracy of the ROI placement (Figure 1). From this review, six study subjects (four controls and two patients) had either one or two misaligned masks (right SN in two controls and one PD patient, right caudate in two controls and one PD patient, left SN in one PD patient and left caudate in one control and one PD patient). The misaligned ROIs were removed from subsequent analysis.

### Extraction of DTI measures for analysis

Following the blinded qualitative assessment, the FMRIB's *fslstats* tool was used to apply the native space masks for each subcortical structure to the voxel wise maps of FA, MD, AD, and RD in each study subject and extract the mean values.

### Statistical analyses

Demographic variables were compared using two-tailed *t*-tests and chi-squared tests with significance was set to *p* < 0.05. Two sided Satterthwaite *T*-test contrasts were used to compare FA, MD, AD, and RD values of right and left deep gray matter structures in all PD patients vs. controls, with significance set to *p* < 0.05. To determine if DTI measures differ between PD subtypes, we used an analysis of variance (ANOVA) with unequal variance and Satterthwaite degrees of freedom to test for a significant group effect across all groups and between each pairwise group comparison (TD vs. Controls, PIGD vs. Controls, and TD vs. PIGD). If the overall ANOVA *F*-test identified any differences among the three groups, then pairwise comparisons were made among the groups using a Tukey-Kramer adjustment for multiple comparisons.

Partial Spearman's correlations by group (all PD patients, TD, and PIGD) and adjusted for age were used to check for post hoc correlations between FA and MD and disease duration (in years), disease stage (H&Y stage), and motor severity (MDS-UPDRS III) for those basal ganglia structures identified as having significant group differences in the ANOVA affecting at least one side. Clinical correlations with AD and RD were not tested in order to limit the overall number of correlations tested and to avoid multicollinearity issues as these measures were strongly correlated with MD in our control dataset (MD to AD with mean rho: 0.93 ± 0.065; and MD to RD with mean rho: 0.98 ± 0.017).

As patients with PD generally have an asymmetric onset and progression of their symptoms, we repeated our statistical analysis using an approach to try to control for the predominant symptom side and assess whether aligning the presumed more affected sides in patients altered our findings. To do this, patients who had their left side body (right brain) affected at onset had their brain data flipped right to left such that the left brain represented the presumably more affected side across PD patients. Similar statistical tests were then applied as described above with the exception that an average of the left and right sides was used for the healthy control values when making comparisons between PD patients and healthy controls.

## Results

### Subject characteristics

Healthy controls (*N* = 20) and PD patients (*N* = 21) were closely matched in age (61.1 ± 9.0 vs. 61.1 ± 7.7, *p* = *0.97*) and gender (11M:9F vs. 12M:9F, *p* = 0.89). Patient demographics broken down by motor subtype are shown in Table 1. Disease severity ranged from mild to moderate (mean H&Y = 2.2 ± 0.7). No significant differences in motor severity were seen, as per MDS-UPDRS III, in either “off” or “on” medication states between TD and PIGD. No significant differences were identified between the groups in the MoCA (TD: 27.8 ± 1.5, PIGD: 27.6 ± 1.7; *p* = 0.79), BDI (TD: 8.5 ± 5.3, PIGD: 7.8 ± 6.4; *p* = 0.79), and NPI (TD: 2.2 ± 1.8, PIGD: 3.1 ± 3.4; *p* = 0.46).

**Table 1:** **Patient demographic data**.

	**Parkinson Disease Patients**			
	**All *N* = 21**	**TD *N* = 12**	**PIGD *N* = 9**	***p*-value (TD vs. PIGD)**
Age, y	61.1 ± 7.7	60.9 ± 8.3	61.4 ± 7.4	0.88
Gender (M:F)	12:9	9:3	3:6	0.06
Initial side affected (L:R)	9:12	4:8	5:4	0.33
Disease duration, y	5.5 ± 3.4	5.2 ± 2.7	5.9 ± 4.3	0.64
Disease stage (H&Y)	2.2 ± 0.7	2.0 ± 0.4	2.5 ± 0.9	0.08
MDS-UPDRS I	9.6 ± 4.5	9.1 ± 4.3	10.2 ± 5.0	0.58
MDS-UPDRS II	9.8 ± 6.7	7.3 ± 3.5	13.1 ± 8.6	0.05
MDS-UPDRS III				
Off	31.4 ± 10.0	33.5 ± 10.0	28.7 ± 10.0	0.29
On	22.2 ± 9.6	23.5 ± 8.4	20.6 ± 11.4	0.50
MDS-UPDRS IV	1.9 ± 2.1	1.8 ± 1.8	2.0 ± 2.6	0.80
LEDD, mg	505.4 ± 366.7	537.3 ± 384.0	462.9 ± 360.4	0.66
MoCA	27.7 ± 1.5	27.8 ± 1.5	27.6 ± 1.7	0.78
BDI	8.2 ± 5.7	8.5 ± 5.3	7.8 ± 6.4	0.78
NPI-Severity	2.6 ± 3.4	2.2 ± 1.8	3.1 ± 3.4	0.42

*Values shown as mean ± sd except where indicated. p-values calculated using two-tailed independent sample t-tests except for gender and initial side affected differences tested using chi-square test. BDI, Beck Depression Inventory; H&Y, Hoehn & Yahr score; LEDD, Levodopa equivalent daily dose; MDS-UPDRS, Movement Disorder Society-Unified Parkinson Disease Rating Scale; MoCA, Montreal Cognitive Assessment; NPI, Neuropsychiatric Inventory; PIGD, Postural Instability Gait Difficulty; TD, Tremor Dominant*.

### Comparison of PD patients to healthy controls

PD patients had reduced FA in the right SN (*p* = 0.020), and increased MD in the SN (left: *p* = 0.039*;* right: *p* = 0.020) and globus pallidus (left: *p* = 0.020*;* right: *p* = 0.049) when compared to controls (Table 2). Additionally, PD patients had increased RD in the right SN (*p* = 0.022) and bilateral globus pallidus (left: *p* = 0.026*;* right: *p* = 0.049). No significant differences were seen for the caudate or putamen. When adjusting data for predominant motor symptom side, reduced FA persisted in the SN ipsilateral to the more affected body side (*p* = 0.034) and a difference in FA in the SN contralateral to the more affected body side came close to being significant (*p* = 0.007). In addition, the findings of increased MD in the SN (contralateral to the more affected side: *p* = 0.027; ipsilateral to the more affected side: *p* = 0.008) and globus pallidus (contralateral to the more affected side: *p* = 0.023; ipsilateral to the more affected side: *p* = 0.021), as well as increased RD in the SN ipsilateral to the more affected side (*p* = 0.013) and bilateral globus pallidus (contralateral to the more affected side: *p* = 0.025; ipsilateral to the more affected side: *p* = 0.025), were still present.

**Table 2:** **DTI measures across study subjects**.

		**Study Subjects**				**PD vs. HC**
		**Controls**	**PD**	**TD**	**PIGD**	***p*-values (side-adjusted)**
**FA VALUES**						
Substantia Nigra	L	0.63 ± 0.07	0.62 ± 0.06	0.65 ± 0.06	0.57 ± 0.05	0.4501 (0.0700)
	R	0.67 ± 0.06	0.62 ± 0.07	0.63 ± 0.06	0.60 ± 0.08	**0.0198 (0.0337)**
Globus Pallidus	L	0.49 ± 0.10	0.44 ± 0.08	0.44 ± 0.08	0.45 ± 0.09	0.1067 (0.0794)
	R	0.48 ± 0.08	0.44 ± 0.06	0.42 ± 0.07	0.45 ± 0.06	0.1009 (0.1022)
Caudate	L	0.23 ± 0.05	0.24 ± 0.07	0.24 ± 0.08	0.23 ±0.07	0.5538 (0.5424)
	R	0.22 ± 0.05	0.23 ± 0.05	0.23 ± 0.04	0.24 ± 0.07	0.4052 (0.5340)
Putamen	L	0.31 ± 0.08	0.30 ± 0.06	0.29 ± 0.06	0.30 ± 0.08	0.4109 (0.4849)
	R	0.30 ± 0.04	0.29 ± 0.05	0.28 ± 0.04	0.30 ± 0.06	0.6879 (0.4881)
**MD VALUES (× 10^−3^ mm^2^/s)**						
Substantia Nigra	L	0.73 ± 0.23	0.88 ± 0.2	0.79 ± 0.16	0.98 ± 0.20	**0.0385 (0.0273)**
	R	0.77 ± 0.20	0.94 ± 0.22	0.90 ± 0.19	0.99 ± 0.25	**0.0204 (0.0083)**
Globus Pallidus	L	0.70 ± 0.12	0.79 ± 0.11	0.77 ± 0.11	0.81 ± 0.10	**0.0197 (0.0225)**
	R	0.74 ± 0.13	0.81 ± 0.10	0.81 ± 0.10	0.82 ± 0.10	**0.0492 (0.0211)**
Caudate	L	1.06 ± 0.57	0.98 ± 0.61	0.95 ± 0.54	1.02 ± 0.73	0.6368 (0.6726)
	R	1.10 ± 0.55	0.99 ± 0.50	0.95 ± 0.41	1.05 ± 0.64	0.4904 (0.6880)
Putamen	L	0.75 ± 0.13	0.78 ± 0.05	0.77 ± 0.00	0.79 ± 0.00	0.3282 (0.6503)
	R	0.79 ± 0.07	0.79 ± 0.04	0.79 ± 0.00	0.78 ± 0.00	0.7880 (0.5120)
**AD VALUES (× 10^−3^ mm^2^/s)**						
Substantia Nigra	L	1.33 ±0.28	1.50 ± 0.31	1.48 ± 0.19	1.52 ± 0.42	0.0901 (0.1171)
	R	1.46 ± 0.28	1.61 ± 0.34	1.62 ± 0.24	1.59 ± 0.45	0.1431 (0.0614)
Globus Pallidus	L	1.06 ± 0.12	1.14 ± 0.13	1.11 ± 0.13	1.18 ± 0.13	0.0526 (0.0762)
	R	1.10 ± 0.15	1.17 ± 0.13	1.15 ± 0.13	1.21 ± 0.14	0.1115 (0.0579)
Caudate	L	1.25 ± 0.60	1.17 ± 0.63	1.14 ± 0.57	1.21 ± 0.76	0.6658 (0.6907)
	R	1.30 ± 0.58	1.17 ± 0.53	1.13 ± 0.44	1.24 ± 0.67	0.4925 (0.7126)
Putamen	L	0.98 ± 0.12	1.01 ± 0.06	1.00 ± 0.00	1.03 ± 0.00	0.3384 (0.8304)
	R	1.03 ± 0.10	1.02 ± 0.07	1.01 ± 0.00	1.02 ± 0.00	0.6702 (0.6418)
**RD VALUES (× 10^−3^ mm^2^/s)**						
Substantia Nigra	L	0.43 ± 0.21	0.55 ± 0.19	0.45 ± 0.15	0.66 ± 0.18	0.0790 **(0.0375)**
	R	0.43 ± 0.18	0.58 ± 0.21	0.53 ± 0.17	0.64 ± 0.25	**0.0221 (0.0123)**
Globus Pallidus	L	0.52 ± 0.14	0.61 ± 0.11	0.60 ± 0.11	0.62 ± 0.12	**0.0263 (0.0245)**
	R	0.56 ± 0.13	0.63 ± 0.10	0.64 ± 0.10	0.63 ± 0.10	**0.0485 (0.0254)**
Caudate	L	0.97 ± 0.55	0.88 ± 0.60	0.85 ± 0.53	0.93 ± 0.71	0.6210 (0.6635)
	R	1.01 ± 0.53	0.89 ± 0.49	0.85 ± 0.39	0.95 ± 0.62	0.4908 (0.6750)
Putamen	L	0.63 ± 0.14	0.67 ± 0.06	0.66 ± 0.00	0.67 ± 0.00	0.3625 (0.6028)
	R	0.67 ± 0.07	0.67 ± 0.04	0.68 ± 0.00	0.66 ± 0.00	0.9133 (0.4923)

*Values shown as mean ± sd except where indicated. p-values calculated using two-tailed independent samples t-tests. Significant p-values, p < 0.05, are shown in bold. See text for details on side adjustment analysis in PD patients. For side-adjusted values, L is contralateral to the more affected body side and R is ipsilateral to the more affected body side. AD, Axial Diffusivity; FA, Fractional Anisotropy; MD, Mean Diffusivity; PD, Parkinson disease; PIGD, Postural Instability/Gait Difficulty; RD, Radial Diffusivity; TD, Tremor Dominant*.

### Comparison of TD and PIGD motor subtypes and healthy controls

**Figure 2:**
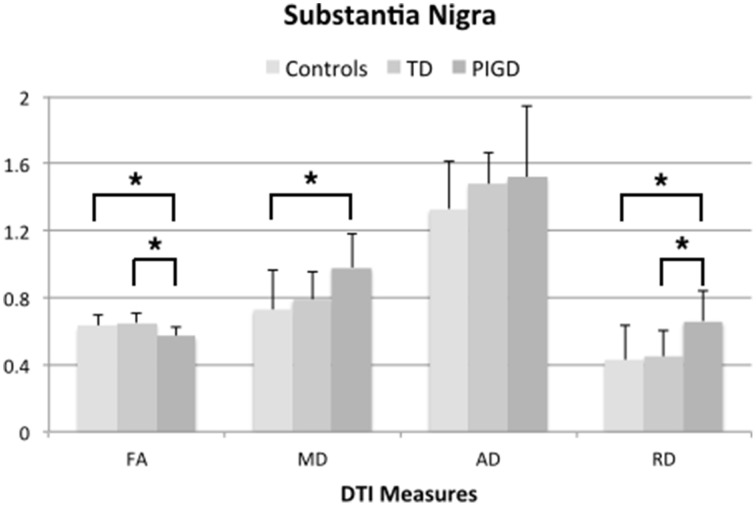
DTI measures from left substantia nigra across groups showing significant differences (^*^p < 0.05, corrected for multiple comparisons using Tukey-Kramer adjustment).

ANOVA testing across the TD and PIGD motor subtypes and healthy controls revealed significant differences in FA (*F* = 6.3, *p* = 0.007), MD (*F* = 4.4, *p* = 0.029), and RD (*F* = 5.2,*p* = 0.017) in the left SN (Table 3). Pairwise comparisons showed that these differences were driven by the PIGD subtype and that no significant differences were seen between TD patients and controls (Table 3, Figure 2). Similar group differences were seen in the right SN, but these did not reach significance. No significant differences across groups were seen for the caudate, putamen, or globus pallidus. When accounting for predominant motor symptom side, similar differences in DTI measures across groups were seen in the SN ipsilateral to the more affected side (FA: *F* = 6.6, *p* = 0.008; MD: *F* = 5.3, *p* = 0.018; RD: *F* = 5.5, *p* = 0.016). A difference across groups in MD within the globus pallidus contralateral to the more affected side also reached our significance threshold (*F* = 4.6, *p* = 0.021). For each of these findings, pairwise comparisons showed revealed that the group differences were driven by the PIGD subtype (Table 3).

**Table 3:** **Pairwise group comparisons of DTI measures in bilateral basal ganglia**.

**FA values**		**ANOVA**		**Pairwise comparisons**		
		***F*/*p*-values**	***F*/*p* (side-adjusted)**	***p*****-values (side-adjusted)**		
				**TD vs. HC**	**PIGD vs. HC**	**TD vs. PIGD**
Substantia Nigra	L	**6.30/0.0073**	2.07/0.1612	0.6867 (0.5991)	**0.0351** (0.1561)	**0.0096** (0.4877)
	R	3.01/0.0808	**6.55**/**0.0078**	0.2270 (0.8649)	0.1094 (**0.0077**)	0.6108 (**0.0344**)
Globus Pallidus	L	1.37/0.2795	1.61/0.2278	–	–	–
	R	1.76/0.1966	2.37/0.1218	–	–	–
Caudate	L	0.21/0.8154	0.20/0.8240	–	–	
	R	0.35/0.7126	0.19/0.8274	–	–	–
Putamen	L	0.48/0.6299	0.35/0.7113	–	–	–
	R	0.63/0.5459	0.85/0.4455	–	–	
**MD VALUES (× 10^−3^ mm^2^/s)**						
Substantia Nigra	L	**4.43/0.0285**	3.04/0.0770	0.6624 (0.3670)	**0.0233** (0.0767)	0.0912 (0.4639)
	R	3.05/0.0772	**5.25/0.0176**	0.1127 (0.2854)	0.0518 (**0.0150**)	0.6343 (0.1956)
Globus Pallidus	L	3.45/0.0524	**4.61/0.0210**	0.2282 (0.3910)	0.0505 (**0.0172**)	0.6954 (0.4160)
	R	2.08/0.1532	2.89/0.0842	–	–	–
Caudate	L	0.17/0.8431	0.19/0.8253	–	–	–
	R	0.42/0.667	0.15/0.8597	–	–	–
Putamen	L	0.74/0.4852	0.53/0.5928	–	–	–
	R	0.07/0.9339	0.33/0.7223	–	–	–
**AD VALUES(× 10^−3^ mm^2^/s)**						
Substantia Nigra	L	1.66/0.2269	1.46/0.2671	–	–	–
	R	1.43/0.273	2.17/0.1519	–	–	–
Globus Pallidus	L	2.98/0.0771	3.24/0.0624	–	–	–
	R	1.65/0.2206	2.00/0.1668	–	–	–
Caudate	L	0.14/0.8714	0.18/0.8387	–	–	–
	R	0.42/0.6641	0.13/0.8792	–	–	–
Putamen	L	1.3/0.2884	0.80/0.4630	–	–	–
	R	0.21/0.8089	0.20/0.8230	–	–	–
**RD VALUES(× 10^−3^ mm^2^/s**)						
Substantia Nigra	L	**5.17/0.0173**	2.82/0.0906	0.9396 (0.4693)	**0.0194** (0.0859)	**0.0358** (0.4109)
	R	2.95/0.0844	**5.46**/**0.0161**	0.2738 (0.4652)	0.1081 (**0.0137**)	0.5739 (0.1003)
Globus Pallidus	L	2.69/0.0943	3.43/0.0518	–	–	–
	R	2.06/0.1561	3.12/0.0710	–	–	–
Caudate	L	0.19/0.8274	0.20/0.8188	–	–	–
	R	0.41/0.6693	0.17/0.8486	–	–	–
Putamen	L	0.44/0.6506	0.20/0.8221	–	–	–
	R	0.3/0.7411	0.71/0.5032	–	–	–

*F-and p-values calculated using ANOVA with unequal variance across controls, TD and PIGD groups. Pairwise comparisons were adjusted for multiple comparisons using a Tukey-Kramer correction. Significant p-values, p < 0.05 (corrected), are shown in bold. See text for details on side adjustment analysis in PD patients. For side-adjusted values, L is contralateral to the more affected body side and R is ipsilateral to the more affected body side. AD, Axial Diffusivity; FA, Fractional Anisotropy; MD, Mean Diffusivity; PIGD, Postural Instability/Gait Difficulty; RD, Radial Diffusivity; TD, Tremor Dominant*.

### Clinical correlations with DTI measures

Across all PD patients, H&Y disease stage showed a negative correlation with FA in the left SN (*r* = −0.482, *p* = 0.037). Within TD, there were no significant correlations between DTI measures in the SN or globus pallidus and clinical measures. In the PIGD group, however, MD in the globus pallidus showed a positive correlation with H&Y disease stage (right: *r* = 0.764, *p* = 0.027) and MDS-UPDRS III scores (left: MD: *r* = 0.814, *p* = 0.014). After adjusting our data for the predominant motor symptom side, no significant correlations were found between FA in the SN and clinical assessments in the PD patients. In the PIGD group, MD in the globus pallidus ipsilateral to the more affected side was positively correlated with MDS-UPDRS III scores (*r* = 0.785, *p* = 0.021). No significant correlations were seen between any of DTI measures and disease duration either across or within subtypes.

## Discussion

Our results build upon prior reports of reduced FA and increased diffusivity measures within the SN in PD by demonstrating that such changes are much more prominent in PIGD patients than TD patients. Furthermore, increased MD in the globus pallidus in PD patients was also driven by the PIGD patients, and these changes correlated with disease stage and motor severity in patients with this motor subtype.

Decreased FA along with increased MD and RD within the SN have been previously reported in a 1-methyl-4-phenyl-1,2,3,6-tetrahydropyridine (MPTP)-induced murine PD model (Boska et al., 2007). In this study, RD and MD values correlated with the degree of depletion of the dopaminergic nigrostriatal neurons. In PD patients, decreased FA within the SN have also been detected using DTI. Cochrane and Ebmeier, in a meta-analysis of DTI studies and Parkinsonian syndromes, noted that 7 out of 9 PD studies reported significantly decreased nigral FA values (Cochrane and Ebmeier, 2013) compared to controls. Schwarz et al. in another meta-analysis, described 9 out of 10 studies reporting decreased FA in the SN of PD patients, however, with a wide range of FA values reported (Schwarz et al., 2013). Although our absolute FA values were higher than those obtained from the standard reference DTI atlas in the report by Schwarz et al. they were comparable to a number of prior studies (Vaillancourt et al., 2009; Péran et al., 2010; Du et al., 2012; Zhan et al., 2012).

Vaillacourt et al. recently reported that with accurate placement of SN ROIs, FA in the SN could separate PD patients and controls with 100% sensitivity and specificity (Vaillancourt et al., 2009). Given the neuropathologic hallmark of PD is the loss of nigrostriatal dopaminergic neurons (Rudow et al., 2008), our findings help further support that abnormal diffusivity measures in the SN stem directly from neurodegeneration of nigral neurons. A recent DTI study involving a large population of patients from the Parkinson's Progression Marker Initiative (PPMI) however, failed to duplicate the degree of sensitivity in Vaillacourt's report despite using the same ROI drawing approach (Schuff et al., 2015).

Only a small number of DTI studies to date have reported microstructural abnormalities outside the SN in PD. Increased MD in the globus pallidus, in line with our findings, was reported by Kim et al. in a study using voxel-based Tract-Based Spatial Statistics (TBSS; Kim et al., 2013). In that study, findings also included increased diffusivity in the putamen, caudate, thalamus and multiple white matter tracts, despite absence of FA or MD changes in the SN. Pallidal changes in PD are further supported by a pathologic study by Rajput et al. who measured dopamine concentrations in the basal ganglia in post-mortem fresh brain tissue of PD patients using high performance liquid chromatography with electrochemical detection (Rajput et al., 2008). Decreased levels of dopamine were found both in the internal and external globus pallidus of PD patients in comparison to healthy controls to a degree felt to be significant to impair normal motor function.

We found in our study here that significant group differences in FA, MD, and RD in the SN, as well as in MD in the globus pallidus, were driven by the PIGD motor subtype, suggesting that changes in DTI measures are larger in PD patients with predominant balance and gait impairment and not as prominent in tremor-dominant patients. Only a few studies have specifically compared DTI measures in these PD motor subtypes (Lenfeldt et al., 2013; Chan et al., 2014; Schuff et al., 2015). Lenfeldt et al. reported increased MD in the thalamus of TD patients compared to PIGD (Lenfeldt et al., 2013), raising the possibility that microstructural changes within structures outside the basal ganglia could differ between subtypes. No abnormalities were found in the SN or globus pallidus, however, possibly due to methodological differences in DTI acquisition and analysis as their investigation was performed using a 1.5T MRI scanner and two different DTI protocols (6 and 32 gradient directions) were used.

In a DTI study by Chan et al. (2014), FA within the SN did not significantly differ between PIGD compared to other PD patients and controls. The patients with the PIGD subtype, however, did show significant differences in FA within the transcallosal motor tract that differentiated them from more typical PD patients with tremor. Our evaluation was focused on the basal ganglia and not a whole brain analysis, possibly giving us greater power to detect differences between motor subtypes within the SN. In the recent PPMI-based DTI study noted above, PD patients were dichotomized as tremor dominant or non-tremor dominant and diffusion measures within the SN were evaluated (Schuff et al., 2015). While a tendency toward higher AD and RD values in the SN were seen in the non-tremor dominant patients, no significant FA changes were noted between the two groups. Although this study involved a larger cohort compared to our study, patients with an indeterminate motor subtype were combined with non-tremor dominant patients and subjects were recruited and scanned at 10 different sites potentially leading to greater variability within the data. Furthermore, the analysis focused only on the SN and the ROI placement was performed manually, which may be less reliable than our atlas-based approach.

A post-mortem neuropathologic study by Jellinger et al. helps support our finding that microstructural changes in the SN may differentiate PD motor subtypes. In this study, significant differences in the pattern of distribution of dopaminergic neuronal loss, being predominantly ventrolateral in the SN pars compacta projecting to the dorsal putamen in PIGD and more medial in the SN projecting to the caudate nucleus and anterior putamen in TD (Jellinger, 1999). In the present study we utilized probabilistic maps for automated ROI delineation of basal ganglia structures to minimize human interference and imprecisions from manual placement in each subject. The SN ROI we used was obtained from a probabilistic map generated using an ultra-high resolution Fast Low Angle Shot (FLASH) sequence with 0.5 mm isotropic voxels providing exquisite details to delineate the SN (Keuken et al., 2014). We set a fairly high threshold of up to 50% to the margins of the ROI to ensure that we were well within the confines of the SN. One drawback of this approach is that sub-regions of the SN, such as the rostral and caudal aspects, were not amenable to assessment. Further investigation using improved DTI techniques or a higher field strength MRI will be needed to help answer whether diffusion changes within sub-regions of the SN, as well as within other brainstem structures thought to contribute to postural instability and gait impairment such the pedunculopontine nucleus, underlie the differing motor manifestations in PD.

In our study we found that decreased FA within the SN in PD patients correlated with H&Y stage. Similar findings have been reported in prior DTI studies of PD patients (Chan et al., 2007; Prakash et al., 2012; Zhan et al., 2012; Zhang et al., 2015). By investigating relationships between DTI and clinical measures by PD subtype, we also found that diffusivity changes in the globus pallidus of PIGD patients correlated with both H&Y stage and MDS-UPDRS III scores. When our data were adjusted for side of symptom onset, we no longer saw correlations between DTI measures and H&Y stage, but we continued to see a correlation between MD in the globus pallidus and motor severity as measured by MDS-UPDRS III. These findings suggest that greater structural disruption within the globus pallidus could underlie the more severe postural and gait impairments that define the PIGD subtype. This is supported by the study by Rajput et al. discussed above that found that PIGD patients have a greater reduction of dopamine in their globus pallidus overall when compared to healthy controls, TD patients, and PD patients with indeterminate motor subtype (Rajput et al., 2008).

Additional evidence of the importance of the globus pallidus in the phenotypic expression of PD comes from DBS literature. A meta-analysis of the long-term effects of DBS on motor symptoms conducted by George et al. found that PIGD patients had improvement in posture and gait following stimulation of both the globus pallidus and subthalamic nucleus, but improvement in those patients with subthalamic stimulation was followed by a decline not observed with pallidal stimulation (St George et al., 2010). In contrast, in a recent re-analysis of a large DBS clinical trial taking into account the different motor subtypes (Katz et al., 2015), the authors concluded that while the overall response to DBS of either the globus pallidus or subthalamic nucleus did not significantly differ between motor phenotypes, TD patients may have greater improvement in gait with pallidal DBS. Although these studies provide a hint that the globus pallidus may be particularly important in regards to posture and gait in PD, further research is needed to determine its role in the expression of its different motor phenotypes.

Despite revealing robust differences in DTI measures between PIGD and TD subtypes, our study is limited by a small sample size and so our results should be interpreted with caution. A larger DTI study of PD subtypes is needed to help confirm the validity of our findings and allow more stringent multiple comparison corrections to be applied. In addition, how alterations in FA and diffusivity relate to specific underlying neuropathological changes remains unclear. In a MPTP-induced PD animal model, decreased FA and increased MD, along with increased RD in the SN have been correlated to the degree of depletion of dopaminergic neurons along the nigrostriatal pathways (Boska et al., 2007). Dopamine levels, however, cannot be the only factor contributing to changes in DTI measures in PD as we found no significant differences in the caudate and putamen, which are known to harbor the greatest loss of dopaminergic nigral projections in PD (Rajput et al., 2008).

Changes in morphology of cells such as demonstrated in pathologic studies in humans showing atrophy of the remaining pigmented cells in the SN in PD as opposed to hypertrophic cells seen in normal aging subjects (Rudow et al., 2008) could also account for our DTI findings. Adding complexity to the interpretation of our results, there are likely differences in the degree of damage within sub-regions of the basal ganglia in PD (Jellinger, 1999; Rajput et al., 2008; Eggers et al., 2011). Furthermore, iron accumulation within the SN may have influenced our DTI processing, and due to the presence of iron methods such as quantitative susceptibility mapping could be a more sensitive means to image changes within the SN in PD (Du et al., 2015).

In conclusion, our results support that DTI can detect microstructural alterations in the SN and globus pallidus of PD patients, and suggest that such changes within the SN may be able to differentiate PIGD and TD motor subtypes. In addition, our findings suggest that diffusivity changes in the globus pallidus are particularly relevant to patients with the PIGD motor subtype and may more accurately reflect motor severity and disease stage than changes affecting the SN. Although our findings need to be validated in a larger population, they suggest further investigation into the role of the globus pallidus in PD motor subtypes could lead to a better understanding of the pathophysiology underlying these different phenotypes.

## Author contributions

LN Project conception and design; analysis of imaging data; drafting and revision of manuscript. JH Analysis of imaging data; revision of manuscript. JT Project conception and design; revision of manuscript. ES Project organization and execution; subject recruitment and scanning. SS Statistical analysis; revision of manuscript. BB Project conception, design, and execution; clinical assessments and scanning of subjects; analysis of imaging data; revision of manuscript.

## Funding

This work was supported by NIH/NCATS Colorado CTSI Grant Number KL2 TR001080, the University of Colorado Department of Neurology, and the University of Colorado Center for NeuroScience.

### Conflict of interest statement

The authors declare that the research was conducted in the absence of any commercial or financial relationships that could be construed as a potential conflict of interest.
